# Toll-like receptor 7 deficiency suppresses type 1 diabetes development by modulating B-cell differentiation and function

**DOI:** 10.1038/s41423-020-00590-8

**Published:** 2021-01-11

**Authors:** Juan Huang, Jian Peng, James Alexander Pearson, Georgios Efthimiou, Youjia Hu, Ningwen Tai, Yanpeng Xing, Luyao Zhang, Jianlei Gu, Jianping Jiang, Hongyu Zhao, Zhiguang Zhou, F. Susan Wong, Li Wen

**Affiliations:** 1 National Clinical Research Center for Metabolic Diseases, Key Laboratory of Diabetes Immunology (Central South University), Ministry of Education, and Department of Metabolism and Endocrinology, The Second Xiangya Hospital, Central South University, Changsha, Hunan China; 2 Section of Endocrinology, Department of Internal Medicine, School of Medicine, Yale University, New Haven, CT USA; 3 Division of Infection and Immunity, School of Medicine, Cardiff University, Cardiff, UK; 4 Department of Microbiology, University of Hull, Hull, UK; 5 Department of Gastrointestinal Surgery of the First Hospital of Jilin University, Changchun, Jilin China; 6 Department of Biostatistics, Data Science & Genetics, Yale School of Public Health, New Haven, CT USA

**Keywords:** Type 1 diabetes, Toll-like receptor 7, B cell, Autoimmunity, Toll-like receptors

## Abstract

Innate immunity mediated by Toll-like receptors (TLRs), which can recognize pathogen molecular patterns, plays a critical role in type 1 diabetes development. TLR7 is a pattern recognition receptor that senses single-stranded RNAs from viruses and host tissue cells; however, its role in type 1 diabetes development remains unclear. In our study, we discovered that *Tlr7*-deficient (*Tlr7*^−/−^) nonobese diabetic (NOD) mice, a model of human type 1 diabetes, exhibited a significantly delayed onset and reduced incidence of type 1 diabetes compared with *Tlr7*-sufficient (*Tlr7*^+/+^) NOD mice. Mechanistic investigations showed that *Tlr7* deficiency significantly altered B-cell differentiation and immunoglobulin production. Moreover, *Tlr7*^−/−^ NOD B cells were found to suppress diabetogenic CD4^+^ T-cell responses and protect immunodeficient NOD mice from developing diabetes induced by diabetogenic T cells. In addition, we found that *Tlr7* deficiency suppressed the antigen-presenting functions of B cells and inhibited cytotoxic CD8^+^ T-cell activation by downregulating the expression of both nonclassical and classical MHC class I (MHC-I) molecules on B cells. Our data suggest that TLR7 contributes to type 1 diabetes development by regulating B-cell functions and subsequent interactions with T cells. Therefore, therapeutically targeting TLR7 may prove beneficial for disease protection.

## Introduction

Type 1 diabetes (T1D) is an autoimmune disorder characterized by the destruction of insulin‐producing pancreatic β‐cell mediated by autoreactive immune cells^1^ that involves both innate and adaptive immunity.^2^ Toll-like receptors (TLRs) are a family of pattern recognition receptors that can induce innate immune responses and modulate adaptive immunity and are widely expressed on/in a variety of immune and nonimmune cells.^3^ Most surface-expressed TLRs can recognize microbe-derived lipids, lipoproteins, and proteins; additionally, several intracellular TLRs, including TLR3, TLR7, TLR8, and TLR9, sense bacterial and viral nucleic acids.^4,5^ In addition to roles in immune responses to pathogens, TLRs can modulate the susceptibility to autoimmune diseases, such as systemic lupus erythematosus (SLE)^6,7^ and arthritis.^8^ In SLE-prone MRL/lpr mice, TLR7 promotes anti-nuclear autoantibody generation and disease development, while TLR9 protects against disease.^9^ TLR9 also regulates TLR7-dependent autoantibody production and disease progression in MRL/lpr mice,^10^ suggesting important crosstalk between these TLRs. In T1D studies, *Tlr2* or *Tlr9* deficiency in nonobese diabetic (NOD) mice led to protection from T1D development,^11–14^ whereas *Tlr4* deficiency accelerated disease progression.^15,16^ TLR7, another member of the TLR family, is mainly expressed in immune cells^4,5,17^ and can recognize single-stranded RNA (ssRNA), a common feature of viral genomes.^18^ However, it is unclear what role TLR7 plays in the context of T1D.

Our previous study identified that norovirus, a murine enteric ssRNA virus, activates TLR7 and modulates susceptibility to T1D in NOD mice.^19^ In addition, rotavirus, an enteric double-stranded RNA virus, can also activate TLR7 to promote lymphocyte activation.^20^ Interestingly, the activation of immune cells from NOD mice in response to rotavirus stimulation in vitro was weaker compared with that of immune cells from C57BL/6 mice.^20^ In contrast, in vivo activation of TLR7 in a diabetogenic CD8 T-cell receptor (TCR) transgenic NY8.3 NOD mouse model accelerated diabetes development.^21^ To ascertain the role of TLR7 in mediating the susceptibility to T1D, we investigated the TLR7-dependent modulation of the immune system that may alter the susceptibility to T1D by using *Tlr7*-deficient (*Tlr7*^−/−^) NOD mice.

## Materials and methods

### Mice

All the mice used in this study were kept in specific pathogen-free conditions with a 12-h dark/light cycle at the Yale University animal facility. NOD/Caj mice were originally obtained from the Jackson Laboratory and have been maintained at Yale University. *Tlr7*^−/−^ C57BL/6 breeders, which were kindly provided by Prof. Richard Flavell (Yale University),^22^ were backcrossed to the NOD/Caj genetic background for 12 generations. The purity of the NOD genetic background was examined by mouse genome scan using an Illumina SNP chip (DartMouse). BDC 2.5 CD4^+^ TCR transgenic NOD mice, NY8.3 CD8^+^ TCR transgenic mice and *Rag*-deficient (*Rag*^−/−^) NOD mice were originally obtained from the Jackson Laboratory and have been maintained at Yale University. The use of the animals in this study was approved by the Institutional Animal Care and Use Committee of Yale University.

### Natural history of diabetes development

*Tlr7*^−/−^ NOD mice and *Tlr7*^+/+^ NOD mice (wild-type NOD mice) were observed for spontaneous diabetes development by screening for glycosuria weekly for 40 weeks. Diabetes was confirmed by a blood glucose concentration ≥ 250 mg/dl (13.9 mmol/l).

### Insulitis score

Mice were dissected, and the pancreata were collected during the prediabetic stage (10–12 weeks, female). Pancreatic tissues were fixed in 10% neutral formalin buffer and embedded in paraffin. The paraffin-embedded pancreata were then sectioned at a thickness of 5 μm and stained with hematoxylin and eosin (H&E). Insulitis was scored under a light microscope using the following grading scale: 0, no infiltration; 1, <25% infiltration of the islets; 2, 25–50% infiltration of the islets; and 3, >50% infiltration of the islets.

### Cell purification

CD4^+^ T cells and CD8^+^ T cells were purified from the spleen of 8-week-old female *Tlr7*^−/−^ NOD mice and *Tlr7*^+/+^ NOD mice by negative selection with magnetic beads, according to the manufacturer’s instructions (QIAGEN). CD4^+^ T cells were purified by removing CD8^+^ T cells (clone: T1B105), MHC class II^+^ cells (clone: 10.2.16), and B cells (anti-mouse IgM and IgG) using mAb hybridoma supernatants, followed by magnetic bead separation (conjugated with goat anti-rat IgG). CD8^+^ T cells were purified by removing CD4^+^ T cells (clone: GK1.5), MHC class II^+^ cells (clone: 10.2.16), and B cells (anti-mouse IgM and IgG). Splenic B cells were purified with an EasySep^TM^ Mouse B-cell isolation kit purchased from STEMCELL Technologies. The purity was routinely 90–95%, as verified by flow cytometry.

### CFSE-labeled cell proliferation in vivo

Purified splenic CD4^+^ BDC 2.5 T cells were labeled with CFSE and injected *i.v*. (3 × 10^6^/mouse) into 8-week-old female *Tlr7*^−/−^ NOD mice and *Tlr7*^+/+^ NOD mice. Three days later, the recipient mice were sacrificed, and lymphocytes were collected from the spleen, pancreatic lymph node (PLN), and mesenteric lymph node (MLN) for flow cytometric analysis of the proliferative division of CFSE-labeled cells.

### T–B-cell interaction assay

Splenic B cells were purified from 8-week-old female *Tlr7*^−/−^ NOD mice and *Tlr7*^+/+^ NOD mice. Purified B cells (1 × 10^5^ cells/well) were treated with mitomycin-C (Sigma) and cocultured with bead-purified splenic CD4^+^ T cells from *Tlr7*^−/−^ NOD mice and *Tlr7*^+/+^ NOD mice (T/B = 1:1) in a 96-well plate with different concentrations of an anti-CD3 mAb (2C11 hybridoma supernatant) at 37 °C in a 5% CO_2_ incubator for 48 h. The proliferation of CD4^+^ T cells was determined by measuring ^3^H-thymidine incorporation for 18 h after the 48-h culture with a β-counter.

### Transwell cell culture assay

Transwell inserts (Fisher) containing 6.25 × 10^5^ purified B cells in 0.5 ml complete medium were placed in a 24-well plate with an equal number of bead-purified CD4^+^ T cells in 0.5 ml medium with an anti-CD28 antibody (1 µg/ml) and different concentrations of an anti-CD3 antibody (2C11 hybridoma supernatant). The cells were incubated at 37 °C in a 5% CO_2_ incubator for 48 h, and the proliferation of purified CD4^+^ T cells in the lower chamber was assessed by measuring ^3^H-thymidine incorporation 18 h later.

### Adoptive cell transfer experiment

Purified splenic CD4^+^ T cells (7 × 10^6^) from diabetic wild-type NOD mice together with purified splenic B cells (7 × 10^6^) from nondiabetic 8-week-old female *Tlr7*^−/−^ NOD mice and *Tlr7*^+/+^ NOD mice were adoptively transferred (*i.v.*) into *Rag*^−/−^ NOD mice (4–5 weeks old). The recipient mice were monitored for diabetes development after adoptive transfer by screening for glycosuria weekly and confirmed to be diabetic by a blood glucose level greater than 250 mg/dl.

### Antigen presentation and blocking assay

Purified splenic B cells (1 × 10^5^ cells/well) from 8-week-old female *Tlr7*^−/−^ NOD mice or *Tlr7*^+/+^ NOD mice were treated with mitomycin-C prior to coculture with NY8.3 CD8^+^ T cells (1 × 10^5^ cells/well) in the presence of 200 ng/ml islet-specific glucose-6-phosphatase catalytic subunit-related protein (IGRP) peptide (IGRP_206-214_) for 48 h. For MHC-I blocking, B cells were cultured in the absence or presence of different concentrations of anti-classical MHC-I (H2-K^d^) and/or anti-nonclassical MHC-I (Qa2) mAbs. The proliferation of CD8^+^ T cells was determined with a ^3^H-thymidine incorporation assay performed 18 h later with measurement on a β-counter.

### Microarray analysis

RNA was extracted from purified splenic B cells from *Tlr7*^−/−^ NOD mice and *Tlr7*^+/+^ NOD mice (8-week-old females), and cRNA synthesis and whole-genome microarray analysis were performed at the Yale Center for Genomic Analysis (Yale University). GeneChip^®^ WT Plus Reagent Kits (Thermo Fisher Scientific) were used for sample preparation and ss-cDNA generation. Total RNA (150 ng) was used for input. Affymetrix GeneChip Mouse Gene 2.0 ST arrays were washed using the GeneChip^®^ Fluidics Station 450 and scanned with the GeneChip Scanner 3000. All the reactions and hybridizations were carried out according to the manufacturer’s protocol.

### Cell staining and flow cytometry analysis

Single-cell suspensions (10^6^) from different mouse lymphoid tissues, including the spleen and lymph nodes, were incubated with an Fc blocker (clone: 2.4G2) at room temperature for 20 min before cell-surface staining. For intracellular cytokine (ICC) staining, cells were incubated at 37 °C for 4 h in the presence of 50 ng/ml PMA (Sigma), 500 ng/ml ionomycin (Sigma) and 1 μl of Golgi PlugTM (BD Bioscience), followed by incubation with monoclonal antibodies specific for surface molecules. ICC staining was then conducted following fixation (20 min, RT) and permeabilization using buffers from Tonbo Biosciences. Cells were stained with antibodies specific for the following surface and intracellular markers: CD45 (clone: 30-F11), TCRβ (clone: H57-597), CD4 (clone: GK1.5), CD8 (clone: 53-6.7), CD19 (clone: 6D5), CD21 (clone: 7E9), CD23 (clone: B3B4), GL-7 (clone: GL-7), IgM (clone: RMM-1), IgD (clone: RB6-8C5), IgG1 (clone: RMG1-1), IgG2a (clone: RMG2a-62), IgG2b (clone: RMG2b-1), TNF-α (clone: MP6-XT22), IL-6 (clone: MP5-20F3), IL-17A (clone: TC11-18H10.1), and IFN-γ (clone: XMG1.2). The stained cells (1–3 × 10^5^ immune cells) were analyzed on a BD LSRII flow cytometer, and results were analyzed with FlowJo 8.8.6 (TreeStar).

### Insulin autoantibody and total immunoglobulin detection

Serum samples collected from 8-week-old female *Tlr7*^−/−^ NOD mice and *Tlr7*^+/+^ NOD mice were tested for anti-insulin autoantibodies by ELISA. Plates were coated with human insulin (4 μg/ml, Lilly) overnight. After washing and blocking (1 h, room temperature, 1% BSA in PBS), diluted (1:100) serum samples were tested for total (Ig) anti-insulin autoantibodies and different isotypes of anti-insulin autoantibodies with Alkaline phosphatase-conjugated (AP-conjugated) goat anti-mouse IgH+L, IgM, IgG, IgA, IgG1, IgG2a, IgG2b, IgG3 and a phosphate substrate. The enzymatic reaction was stopped with NaOH, and the plates were read with a microplate spectrophotometer (Perkin Elmer, Waltham, MA, USA) at OD 405 nm. Different isotypes of total serum immunoglobulins were also measured. Briefly, the wells of a 96-well plate were coated with samples or standards overnight. After washing and blocking (1 h, room temperature, 1% BSA in PBS), the plates were then incubated with AP-conjugated goat anti-mouse IgG1, IgG2a, or IgG2b (2 h, room temperature). The samples were subsequently washed, and the substrate PNPP (Sigma) was added. The reaction was stopped by adding 1 M NaOH, and the samples were analyzed on a microplate spectrophotometer at 405 nm (OD).

### Cytokine ELISA

Serum and/or supernatant concentrations of TNF-α, IFN-γ, IL-17A, IL-6, and IL-10 were measured using ELISA kits according to the manufacturer’s instructions (BioLegend). The TGF-β concentration in supernatants was measured using an ELISA kit purchased from R&D Systems.

### Antibodies and reagents

The fluorochrome-conjugated monoclonal antibodies used in this study were purchased from BioLegend or eBioscience. The supernatants of different monoclonal antibody (mAb) hybridomas were provided by the late Charles Janeway (Yale University). Magnetic beads conjugated with goat anti-mouse IgG, goat anti-mouse IgM, or goat anti-rat IgG were purchased from QIAGEN. RPMI-1640 medium and heat-inactivated FCS were purchased from Invitrogen and Gemini, respectively. The anti-H2-K^d^ mAb was affinity purified from a hybridoma (clone: HB159) supernatant. The anti-Qa2 mAb was purchased from BioLegend (clone: 659H1-9-9). AP-conjugated goat anti-mouse IgH+L, IgG, IgA, IgM, IgG1, IgG2a, and IgG2b for ELISA were purchased from Southern Biotechnology.

### Data analysis

Statistical analysis was performed using GraphPad Prism software version 8.0 (GraphPad Software, San Diego, CA, US). Diabetes incidence was compared using the log-rank test for survival. Insulitis scores were analyzed using a Chi-square test. In vitro assays were analyzed with a two-tailed Student’s *t* test (if the data were normally distributed), a two-tailed Mann–Whitney test (if the data were not normally distributed), multiple *t* tests with the Bonferroni correction, or two-way ANOVA. The *P* value and statistical analysis for each experiment are reported in the figure legends. *P* < 0.05 was considered significant.

## Results

### *Tlr7* deficiency suppresses T1D development in NOD mice

**Fig. 1:**
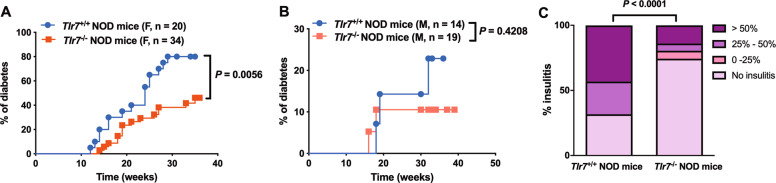
Blockade of *Tlr7* delayed the onset and reduced the development of type1 diabetes. **A** Natural history of type 1 diabetes development in female *Tlr7*^*+/+*^ NOD mice (*n* = 20) and *Tlr7*^−/−^ NOD littermates (*n* = 34). Diabetes development was monitored by weekly glycosuria testing and confirmed by a blood glucose level over 250 mg/dl. **B** Natural history of diabetes development in male *Tlr7*^*+/+*^ NOD mice (*n* = 14) and *Tlr7*^−/−^ NOD littermates (*n* = 19). **C** Insulitis scores of female *Tlr7*^*+/+*^ NOD mice and *Tlr7*^−/−^ NOD mice. Pancreata from nondiabetic female *Tlr7*^*+/+*^ NOD mice and *Tlr7*^−/−^ NOD littermates (*n* = 4–7/group, 10–12 weeks) were fixed in 10% neutral formalin, and insulitis was evaluated under a light microscope after H&E staining. Insulitis was scored in 164–199 islets per group, and the insulitis scores are shown. Data were pooled from at least two independent experiments and analyzed using the log-rank test for survival (**A**, **B**) and a Chi-square test (**C**)

To define the role of TLR7 in T1D development, we generated *Tlr7*^−/−^ NOD mice by backcrossing *Tlr7*^−/−^ C57BL/6 mice with NOD mice for 12 generations. The purity of the NOD genetic background of *Tlr7*^−/−^ NOD mouse was confirmed by a mouse genome scan using an Illumina SNP chip. We first monitored the natural history of T1D development in *Tlr7*^−/−^ NOD mice and *Tlr7*^*+/+*^ NOD littermates. The development of T1D was delayed in female *Tlr7*^−/−^ NOD mice, and the overall disease incidence in these mice was also significantly reduced (Fig. 1A). No significant difference in the incidence of diabetes development was observed between male *Tlr7*^−/−^ NOD mice and male *Tlr7*^+/+^ NOD littermates, although the overall incidence of T1D was much lower in male *Tlr7*^−/−^ NOD mice than in male *Tlr7*^+/+^ NOD littermates (Fig. 1B). To investigate the effect of *Tlr7* ablation on immune cell infiltration in the pancreatic islets, we randomly selected prediabetic female *Tlr7*^−/−^ NOD mice and *Tlr7*^+/+^ NOD littermates (*n* = 4–7 per group, 10–12 weeks old) and harvested the pancreas to examine insulitis. In accordance with the reduction in diabetes development, female *Tlr7*^−/−^ NOD mice displayed significantly less insulitis than their female *Tlr7*^+/+^ NOD counterparts (Fig. 1C). Thus, our data showed that *Tlr7* deficiency limited the infiltration of immune cells into the islets, attenuating the development of T1D in NOD mice.

### *Tlr7* deficiency alters systemic immune responses in NOD mice

**Fig. 2:**
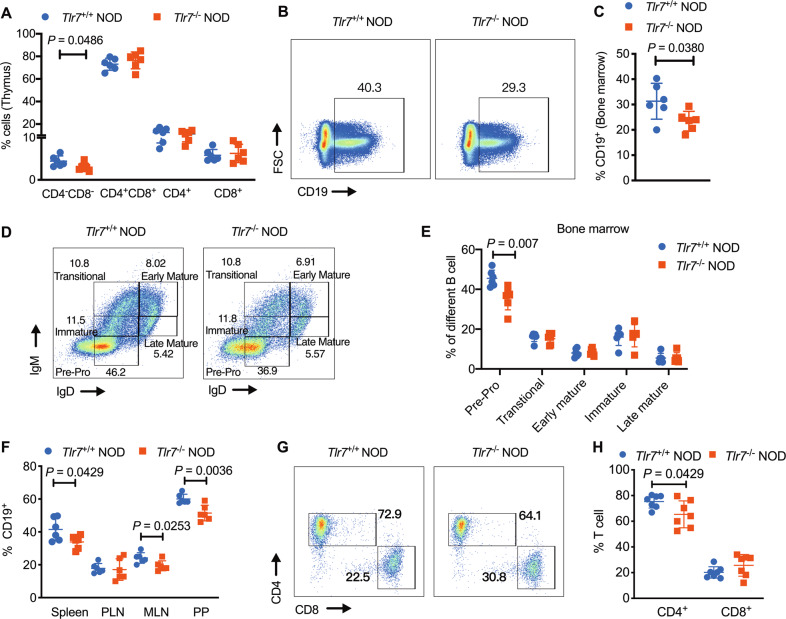
*Tlr7* deficiency altered systemic immune responses in NOD mice. **A** The percentages of thymic CD4^−^CD8^−^, CD4^+^CD8^+^, CD4^+^, and CD8^+^ cells, gated from live single cells from 8-week-old female *Tlr7*^*+/+*^ and *Tlr7*^−/−^ NOD mice. **B**, **C** The proportion of CD19^+^ B cells in the bone marrow of 8-week-old female *Tlr7*^+/+^ and *Tlr7*^−/−^ NOD mice, gated from live single cells. Representative flow cytometric plots (**B**) and the summarized percentage of CD19^+^ B lymphocytes (**C**) are shown. **D**, **E** The percentages of pre–pro B cells, immature B cells, transitional B cells, early mature B cells and late mature B cells in the bone marrow of 8-week-old female *Tlr7*^+/+^ and *Tlr7*^−/−^ NOD mice. Representative flow cytometric plots with further gating of CD19^+^ B cells (D) and the summarized percentages of each B lymphocyte subset (**E**) are shown. **F** The proportions of CD19^+^ B cells in the spleen, PLN, MLN, and PP of 8-week-old female *Tlr7*^+/+^ and *Tlr7*^−/−^ NOD mice, as analyzed by flow cytometry. Cells were gated from the initial population of live single TCR-β^−^ cells. **G**, **H** The percentages of CD4^+^ T cells and CD8^+^ T cells in 8-week-old female *Tlr7*^+/+^ and *Tlr7*^−/−^ NOD mice. Representative flow cytometric plots were gated from the initial population of CD19^−^TCR^−^β^+^ T cells (**G**), and the summarized percentages of CD4^+^ and CD8^+^ T lymphocytes (**H**) are shown. Data pooled from two independent experiments (**A**, **C**, **E**, **F**, **H)** were analyzed by a two-tailed Student’s *t*-test or a two-tailed Mann–Whitney test and are represented as the mean ± SD (*n* = 6–7/group). FSC forward scatter, PLN pancreatic lymph node, MLN mesenteric lymph node, PP Peyer’s patches

To identify the impact of *Tlr7* deficiency on the immune system in NOD mice, we investigated the phenotypes of immune cells in both central lymphoid tissues and peripheral lymphoid tissues. We found that *Tlr7* deficiency did not affect thymocyte development, except for a proportional reduction in the CD4^−^CD8^−^ compartment in *Tlr7*^−/−^ NOD mice (Fig. 2A). However, in the absence of *Tlr7*, NOD mice had a significant reduction in the frequency of bone marrow (BM) B cells (Fig. 2B–C). We also found a reduced proportion of pre–pro B cells in the BM of *Tlr7*-deficient NOD mice (Fig. 2D, E). In line with the reduction in the total B-cell frequency in the BM, the proportions of CD19^+^ B cells in the spleen, MLN, and Peyer’s patches (PP) were also significantly lower in *Tlr7-*deficient NOD mice than in *Tlr7*-sufficient NOD mice, whereas no significant difference was seen in the PLN (Fig. 2F). In addition, the absence of *Tlr7*affected peripheral CD4^+^ T cells but did not obviously impact CD8^+^ T-cell development in PP (Fig. 2G, H). Next, we assessed if *Tlr7* deficiency affects macrophages and dendritic cells (DCs). There were changes in macrophage populations, but we did not find any alterations in the frequencies of conventional DCs (cDCs) and plasmacytoid dendritic cells (pDCs) (Fig. S1A–C). We also measured the type 1 IFN (IFN-α) concentration in the serum. Interestingly, *Tlr7* knockout mice had levels of circulating IFN-α similar to those of their wild-type counterparts (Fig. S1D). Taken together, our results show that *Tlr7* deficiency alters the development of immune cells, particularly B cells, in NOD mice.

### *Tlr7* deficiency alters B-cell differentiation and immune responses

**Fig. 3:**
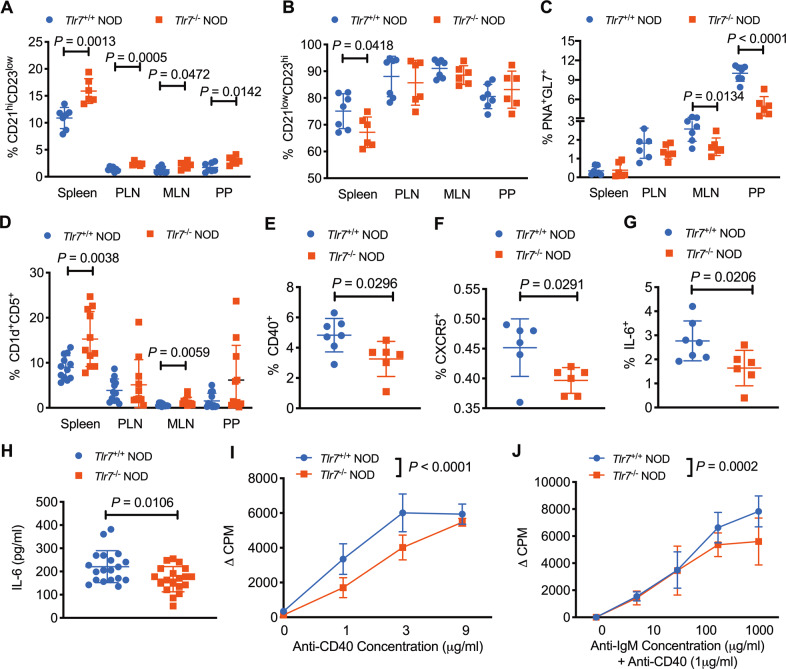
*Tlr7* deficiency affects the differentiation and functional responses of B cells. **A–D** The percentages of B-cell subsets in 8-week-old female *Tlr7*^−/−^ and *Tlr7*^+/+^ NOD mice. CD21^hi^CD23^low^ (**A**, *n* = 6–7/group), CD21^low^CD23^hi^ (**B**, *n* = 6–7/group), PNA^+^GL-7^+^ (**C**, *n* = 6–7/group), and CD1d^+^CD5^+^ (**D**, *n* = 11–12/group), gated from the initial population of TCRβ^−^CD19^+^ B cells. **E**, **F** The proportions of splenic B cells expressing specific activation markers in 8-week-old female *Tlr7*^−/−^ and *Tlr7*^+/+^ NOD mice. CD40^+^ (**E**, *n* = 6–7/group) and CXCR5^+^ (**F**, *n* = 6/group) B cells, gated from the initial population of TCR-β^−^CD19^+^ B cells. **G** The proportion of IL-6-secreting splenic B cells in female *Tlr7*^−/−^ and *Tlr7*^+/+^ NOD mice (*n* = 6–7/group). Cells were gated from the initial population of TCRβ^−^CD19^+^ B cells. **H** Serum IL-6 concentration in 8-week-old female *Tlr7*^−/−^ and *Tlr7*^+/+^ NOD mice (*n* = 19/group). **I**, **J **Proliferation of splenocytes (**I**) or purified splenic B cells (**J**) (5 × 10^4^ cells/well) from 8-week-old female *Tlr7*^−/−^ and *Tlr7*^+/+^ NOD mice. The proliferation of splenocytes cultured with different concentrations of an anti-CD40 antibody (**I**) and purified splenic B cells cultured with different concentrations of an anti-IgM antibody in the presence of the anti-CD40 antibody (**J**) was evaluated. Data pooled from at least two independent experiments (**A**–**H**) are shown as the mean ± SD and were analyzed by a two-tailed Student’s *t*-test or a two-tailed Mann–Whitney test. The data shown in (**I**–**J)** are from one of three representative experiments, all of which showed consistent results and were analyzed using two-way ANOVA. PLN pancreatic lymph node, MLN mesenteric lymph node, PP Peyer’s patches. ∆CPM represents counts per minute after subtracting the background

Next, we determined whether peripheral B-cell subsets are affected by *Tlr7* deficiency and found that the proportion of CD21^hi^CD23^low^ B cells was significantly increased in all the peripheral lymphoid tissues of *Tlr7*^−/−^ NOD mice examined (Fig. 3A), whereas the proportion of CD21^low^CD23^hi^ B cells was reduced, although mostly in the spleen (Fig. 3B). The proportion of germinal center B cells (PNA^+^GL-7^+^) was also significantly reduced in *Tlr7*-deficient NOD mice (MLN and PP) compared with *Tlr7-*sufficient NOD mice (Fig. 3C). Interestingly, the proportions of CD1d^+^CD5^+^ B cells (the common phenotype for Bregs) in the spleen and MLN were significantly increased in *Tlr7*-deficient NOD mice (Fig. 3D). The increased Breg populations were also observed in the PLN and PP, although these increases were not statistically significant (Fig. 3D). In addition, we found that B cells in *Tlr7*^−/−^ NOD mice expressed lower levels of CD40 and CXCR5 than those in *Tlr7*^+/+^ NOD mice (Fig. 3E, F). The expression of IL-6, which is essential for B-cell differentiation, maturation and survival,^23^ was reduced in splenic B cells from *Tlr7*^−/−^ NOD mice (Fig. 3G). The circulating IL-6 concentration was also reduced in *Tlr7*^−/−^ NOD mice (Fig. 3H). Furthermore, we found that splenocytes from *Tlr7*^−/−^ NOD mice displayed attenuated responses to adaptive immune stimulation with an anti-CD40 antibody (Fig. 3I), and importantly, purified splenic B cells showed attenuated responses to stimulation with an anti-IgM antibody in the presence of the anti-CD40 antibody (Fig. 3J). Our results demonstrate that *Tlr7* deficiency in NOD mice not only affects the differentiation of B cells but also decreases the functional responses of these cells.

### *Tlr7* deficiency alters B-cell immunoglobulin production

**Fig. 4:**
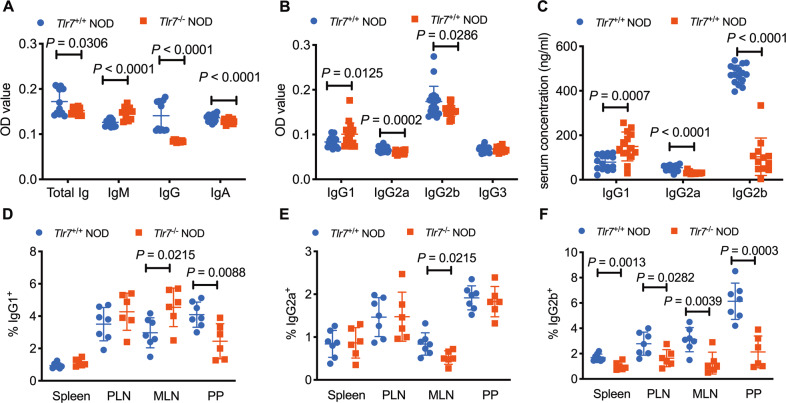
*Tlr7* deficiency affects the production of immunoglobulin by B cells. **A** Serum anti-insulin total Ig, IgM, IgG, and IgA autoantibody levels in 8-week-old female *Tlr7*^−/−^ and *Tlr7*^+/+^ NOD mice. Data are presented as the optical density (OD) measured at 405 nm (*n* = 12–16/group). **B** Serum anti-insulin IgG1, IgG2a, IgG2b, and IgG3 autoantibody levels in female *Tlr7*^−/−^ and *Tlr7*^+/+^ NOD mice (*n* = 12–16/group). **C** Total serum IgG1, IgG2a, and IgG2b antibody concentrations in 8-week-old female *Tlr7*^−/−^ and *Tlr7*^+/+^ NOD mice (*n* = 12–16/group). **D**–**F** Proportions of IgG1^+^ (**D**), IgG2a^+^ (**E**) and IgG2b^+^ (**F**) B cells in the spleen, PLN, MLN, and PP of 8-week-old female *Tlr7*^−/−^ and *Tlr7*^+/+^ NOD mice (*n* = 6–7/group). Cells were gated from the initial population of TCRβ^−^CD19^+^ B cells. Data pooled from two independent experiments (**A**–**F**) were analyzed by a two-tailed Student’s *t*-test or a two-tailed Mann–Whitney test and are shown as the mean ± SD. PLN pancreatic lymph node, MLN mesenteric lymph node, PP Peyer’s patches

Autoantibodies against islet autoantigen(s) have been used as biomarkers for the prediction and diagnosis of T1D, especially in humans.^24^ As *Tlr7* deficiency has strong impacts on B-cell development and functional responses, we hypothesized that the ability of *Tlr7*-deficient B cells to produce (auto)antibodies may also be altered. To test our hypothesis, we measured the levels of anti-insulin antibodies in the serum of prediabetic (8-week-old) *Tlr7*^*+/+*^ NOD mice and *Tlr7*^−/−^ NOD mice. Interestingly, we found that the anti-insulin autoantibody levels in the *Tlr7*^−/−^ NOD mice were indeed much lower than those in the *Tlr7*^*+/+*^ NOD mice, except for the IgM isotype level, which was increased (Fig. 4A). Furthermore, we found that the level of anti-insulin IgG antibodies showed the greatest reduction (Fig. 4A), suggesting that *Tlr7* is important for class switching. We further assessed the levels of different IgG subclasses of anti-insulin antibodies and found that the lower levels of IgG2a and IgG2b likely contributed to the overall decreased level of anti-insulin IgG antibodies (Fig. 4B). Interestingly, the level of anti-insulin IgG1 antibodies was higher in *Tlr7*-deficient mice than in *Tlr7-*sufficient mice (Fig. 4B). Moreover, we found that the total serum antibody concentrations of the IgG subclasses (Fig. 4C) and frequencies of different IgG subclass-poroducing B cells were in accordance with the serum levels of anti-insulin antibodies, particularly for IgG2b (Fig. 4D–F).

### *Tlr7*-deficient B cells attenuate CD4^+^ T-cell functions in a cell contact-dependent manner

**Fig. 5:**
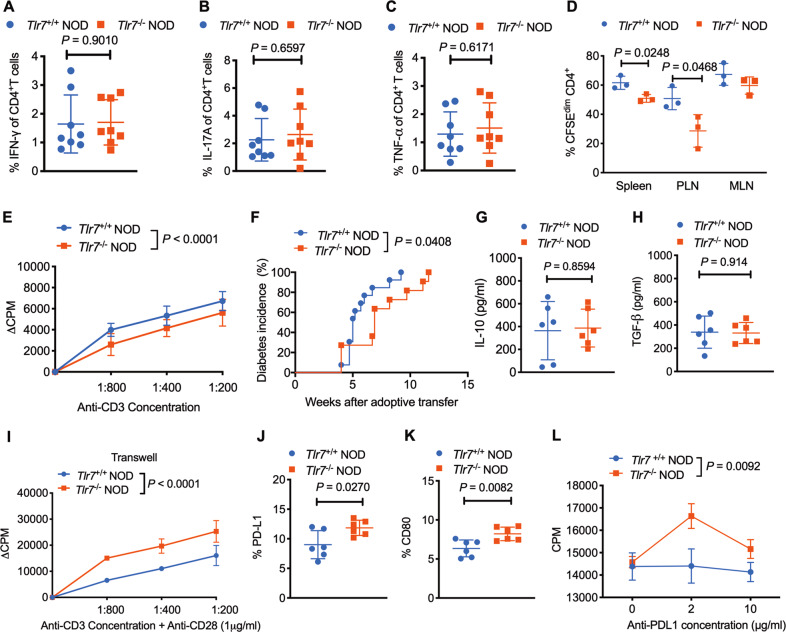
The effect of *Tlr7* deficiency on CD4^+^ T cells in NOD mice. **A–C** Expression levels of IFN-γ, IL-17A, and TNF-α in CD4^+^ T cells after anti-CD3 stimulation (*n*=8/group). IFN-γ (**A**), IL-17A (**B**), and TNF-α (**C**). **D** BDC 2.5 CD4^+^ T-cell proliferation in vivo. CFSE-labeled BDC 2.5 CD4^+^ T cells were injected into 8-week-old female *Tlr7*^−/−^ or *Tlr7*^+/+^ NOD mice, and cell proliferation was evaluated by flow cytometry. **E** Proliferation of *Tlr7*^−/−^ or *Tlr7*^+/+^ NOD CD4^+^ T cells cocultured with purified *Tlr7*^−/−^ NOD B cells in the presence of an anti-CD3 antibody. **F** Purified splenic B cells from *Tlr7*^−/−^ or *Tlr7*^+/+^ NOD mice were cotransferred with purified CD4^+^ T cells from diabetic NOD mice into *Rag*^−/−^ NOD recipient mice, followed by observation for diabetes development. **G**–**H** IL-10 (**G**) and TGF-β (**H**) concentrations in the supernatant of *Tlr7*^−/−^ or *Tlr7*^+/+^ NOD CD4^+^ T cells cocultures with purified splenic *Tlr7*^−/−^ NOD B cells in the presence of an anti-CD3 antibody (*n* = 6/group). **I** Proliferation of *Tlr7*^−/−^ or *Tlr7*^+/+^ NOD CD4^+^ T cells cocultured with purified splenic *Tlr7*^−/−^ NOD B cells in a Transwell system. **J**–**K** Expression of PD-L1 (**J**) and CD80 (**K**) on splenic CD19^+^ B cells. **L** Proliferation of BDC 2.5^+^ NOD CD4^+^ T cells cocultured with purified *Tlr7*^−/−^ or *Tlr7*^+/+^ NOD B cells in the presence of different concentrations of an anti-PD-L1 antibody. The data in (**A**–**C**, **F**–**H**, **J**, **K**) were pooled from two or more independent experiments. The experiments in (**D**, **E**, **I**, **L**) were performed twice, and consistent results were obtained. Data were analyzed using a two-tailed Student’s *t*-test (**A**–**D**, **G**, **H**, **J**, **K**), two-way ANOVA (**E**, **I**, **L**), or the log-rank test for survival (**F**). The data in (**A**–**D**, **G,** **H**, **J**, **K**) are shown as the mean ± SD. PLN pancreatic lymph node, MLN mesenteric lymph node, PP Peyer’s patches. ∆CPM represents counts per minute after subtracting the background

As CD4^+^ T cells are known to play important roles in the destruction of pancreatic β cells and T1D development, we next investigated whether *Tlr7* deficiency affects CD4^+^ T-cell activation and function. Surprisingly, we observed increased IFN-γ-producing CD4^+^ T-cell frequencies in most peripheral lymphoid tissues in *Tlr7-*deficient NOD mice (Fig. S2A). We also observed more IL-17A-producing and TNF-α-producing CD4^+^ T cells in the PP of *Tlr7-*deficient NOD mice than in those of wild-type mice (Fig. S2B, C). When testing for ICC production (i.e., the potential of cells to produce a cytokine), cells were commonly stimulated with PMA and ionomycin, which bypass TCR engagement. To investigate whether TCR engagement actually leads to increased inflammatory cytokine-producing CD4^+^ T-cell frequencies in *Tlr7-*deficient NOD mice, we stimulated total lymphocytes with an anti-CD3 antibody, which directly engages the TCR, in the presence of Golgi Plug^TM^, (omitting the standard treatment with PMA and ionomycin that bypasses TCR signaling) for 4 h. Our results showed that direct TCR engagement through anti-CD3 stimulation did not lead to increased IFN-γ/IL-17A/TNF-α-producing CD4^+^ T-cell frequencies in *Tlr7*^−/−^ NOD mice (Fig. 5A–C). These results suggest that *Tlr7* deficiency does not have direct effects on cytokine-producing CD4^+^ T cells, as T-cell activation in vivo is mediated by TCR engagement via antigen recognition. To further determine whether *Tlr7*-deficient B cells influence diabetogenic CD4^+^ T-cell actions in vivo, we labeled purified BDC 2.5 CD4^+^ T cells (*Tlr7* sufficient) with CFSE and intravenously transferred the cells into *Tlr7*^−/−^ NOD mice or *Tlr7*^*+/+*^ NOD mice. Three days later, we harvested immune cells from different lymphoid tissues of the recipients and evaluated the proliferation of BDC 2.5 CD4^+^ T cells by flow cytometry. It was found that BDC 2.5 CD4^+^ T cells proliferated poorly, particularly in the PLN, in the *Tlr7*-deficient hosts compared with the *Tlr7*-sufficient hosts (Fig. 5D). This suggests that in situ *Tlr7-*deficient antigen-presenting cells (APCs) contribute to the suppression of diabetogenic T-cell expansion in the absence of *Tlr7*.

To test if B cells are responsible for this suppression, we stimulated both *Tlr7*-sufficient CD4^+^ T cells and *Tlr7*-deficient CD4^+^ T cells with an anti-CD3 antibody in the presence of B cells from either *Tlr7*^+/+^ NOD mice or *Tlr7*^−/−^ NOD mice. Interestingly, the CD4^+^ T-cell proliferative response was significantly impaired (Fig. 5E) in the presence of *Tlr7*-deficient B cells, implying that the cross-linking function of *Tlr7*-deficient B cells was less efficient than that of *Tlr7-*sufficient B cells in this context of anti-CD3 antibody stimulation. To further test if *Tlr7*-deficient B cells attenuate diabetes development, we adoptively transferred *Tlr7*-deficient or *Tlr7*-sufficient B cells together with purified CD4^+^ T cells (*Tlr7* sufficient) from diabetic NOD mice into immunodeficient *Rag*^−/−^ NOD recipients. In line with the in vitro coculture results, the *Tlr7*-deficient B cells significantly delayed the diabetes development induced by diabetogenic CD4^+^ T cells in the *Rag*^−/−^ NOD recipients (Fig. 5F).

To investigate whether the suppressive function of *Tlr7*-deficient B cells is mediated by soluble cytokine release, we examined the IL-10 and TGF-β concentrations in the supernatant of the cocultures described above (Fig. 5E). Unexpectedly, there were no significant differences in the IL-10 or TGF-β concentration in the culture supernatants (Fig. 5G, H). Therefore, to determine whether B cells modulate CD4^+^ T cells directly through cell–cell contact, we used a transwell culture system, in which *Tlr7*-deficient B cells were cocultured indirectly with CD4^+^ T cells. Interestingly, when purified B cells from *Tlr7*^−/−^ NOD mice had no contact with *Tlr7*^−/−^ NOD CD4^+^ T cells, we found an increase in CD4^+^ T-cell proliferation (Fig. 5I), which was distinctly different from the suppression seen when CD4^+^ T cells were in direct contact with *Tlr7*^−/−^ NOD B cells (Fig. 5E).

To assess how TLR7 affects the crosstalk between B cells and CD4^+^ T cells, we investigated the expression patterns of costimulatory molecules and chemokine receptors on both B cells and CD4^+^ T cells. We found that the levels of PD-L1 and CD80 were significantly increased on splenic B cells in *Tlr7*^−/−^ NOD mice compared with splenic B cells in *Tlr7*^+/+^ NOD mice (Fig. 5J, K); in contrast, no difference was found in PD-1 on CD4^+^ T cells. To investigate if PD-L1 contributes to the attenuated CD4^+^ T-cell response observed when *Tlr7*-deficient B cells are in direct contact with CD4^+^ T cells, we then blocked PD-L1 with a monoclonal antibody in an antigen-specific T-cell assay. To this end, we cocultured islet autoantigen-specific BDC 2.5 CD4^+^ T cells with mitomycin-C-treated B cells from *Tlr7*^−/−^ NOD mice or *Tlr7*^+/+^ NOD mice, which were used as APCs, in the presence of an antigenic peptide with and without an anti-PD-L1 antibody. Interestingly, we showed that the *Tlr7*^−/−^ NOD B cells but not the *Tlr7*^+/+^ NOD B cells were responsive to blockade with the PD-L1-specific immune checkpoint inhibitor, and hence, the proliferation of BDC 2.5 CD4^+^ T cells was increased (Fig. 5L). Taken together, our data suggest that *Tlr7*^−/−^ B cells in NOD mice restrain CD4^+^ T-cell responses by cell–cell contact, which is potentially mediated through PD-L1.

### *Tlr7* deficiency modulates antigen presentation by B cells to CD8^+^ T cells by regulating nonclassical and classical MHC-I molecule expression

**Fig. 6:**
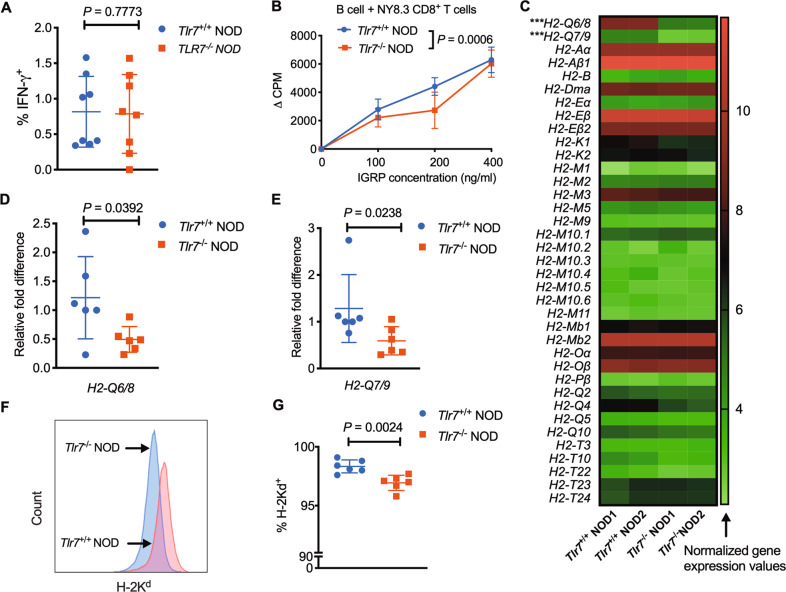
The effect of *Tlr7* deficiency on CD8^+^ T cells in NOD mice. **A** Expression levels of IFN-γ in CD8^+^ T cells after anti-CD3 stimulation (*n* = 8/group). **B** NY8.3 CD8^+^ T-cell proliferation in vitro. NY8.3 CD8^+^ T cells were cultured with purified B cells from either *Tlr7*^+/+^ NOD mice or *Tlr7*^−/−^ NOD mice, in the presence of different concentrations of the IGRP peptide. **C** Microarray results. The heat map shows gene expression levels in purified splenic B cells from 8-week-old female *Tlr7*^*+/+*^ or *Tlr7*^−/−^ NOD mice (*n* = 2/group). Normalized gene expression values are plotted based on the data ranges, where lower gene expression is represented in green, while higher gene expression is represented in red. ^***^P < 0.001 for both *H2-Q6/8* comparisons and *H2-Q7/9* comparisons. **D**–**E **qPCR results for the gene expression of *H2-Q6/8* (**D**) and *H2-Q7/9* (**E**) in purified splenic B cells from 8-week-old female *Tlr7*^*+/+*^ and *Tlr7*^−/−^ NOD mice (*n* = 6/group). **F**–**G** Flow histogram (**F**) and summarized percentage of H2-K^d^ expression (**G**) on splenic CD19^+^ B cells from 8-week-old female *Tlr7*^+/+^ or *Tlr7*^−/−^ NOD mice (*n* = 6/group). The data in (**A**, **D**, **E**, **G**) were pooled from two independent experiments. The experiments in (**B**) were performed twice, and consistent results were obtained. The data were analyzed using a two-tailed Mann–Whitney test (**A**, **E**), two-way ANOVA (**B**), multiple *t*-tests wi*t*h the Bonferroni correction (**C**), or a two-tailed Student’s *t*-test (**D**, **G**). PLN pancreatic lymph node, MLN mesenteric lymph node, PP Peyer’s patches, IGRP islet-specific glucose-6-phosphatase catalytic subunit-related protein. ∆CPM represents counts per minute after subtracting the background

**Fig. 7:**
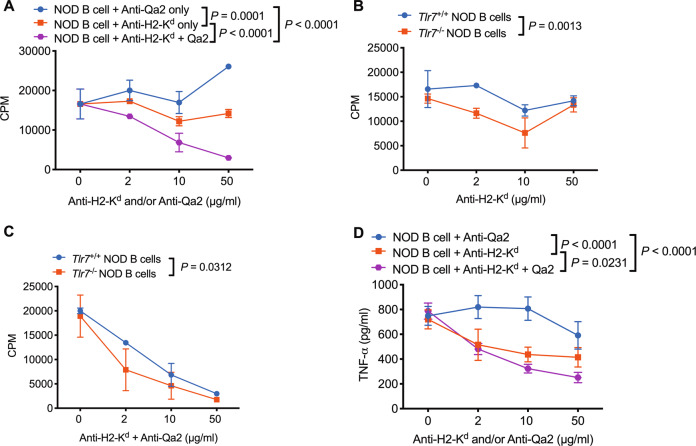
The effect of *Tlr7*-deficient B cells on cytotoxic CD8^+^ T-cell activation in NOD mice. **A** Proliferation of NY8.3 CD8^+^ T cells cocultured with purified splenic wild-type NOD B cells in the presence of 100 ng/ml IGRP and different concentrations of an anti-H2-K^d^ mAb and/or anti-Qa2 mAb. **B**, **C** Proliferation of NY8.3 CD8^+^ T cells cocultured with purified splenic *Tlr7*^+/+^ or *Tlr7*^−/−^ NOD B cells in the presence of 100 ng/ml IGRP and different concentrations of the anti-H2-K^d^ mAb (**B**) or the anti-H2-K^d^ mAb together with the anti-Qa2 mAb (**C**). **D** TNF-α concentration in the supernatant of NY8.3 CD8^+^ T cells cocultured with purified splenic NOD B cells in the presence of 100 ng/ml IGRP and different concentrations of the anti-H2-K^d^ mAb and/or anti-Qa2 mAb (*n* = 6/group). The experiments in (**A**–**C**) were performed twice, and consistent results were obtained. The data in (**D**) were pooled from two independent experiments. Data were analyzed using two-way ANOVA (**A**–**D**)

In addition to CD4^+^ T cells, cytotoxic CD8^+^ T cells play essential roles in β-cell damage and T1D development.^25–29^ Thus, we also investigated the role of *Tlr7* deficiency in regulating CD8^+^ T cells in *Tlr7*^−/−^ NOD mice. Similar to the results for CD4^+^ T cells (Fig. S2A), increased IFN-γ expression was observed in CD8^+^ T cells from the PLN and PP in *Tlr7*^−/−^ NOD mice after stimulation with PMA/ionomycin ex vivo (Fig. S3), but no difference was found if the cells were stimulated with an anti-CD3 antibody (Fig. 6A). However, when we assessed the antigen presentation by B cells to diabetogenic NY8.3 CD8^+^ T cells, our results showed that the antigen-presenting ability of *Tlr7*^−/−^ NOD B cells was significantly impaired, as indicated by reduced NY8.3 CD8^+^ T-cell proliferation (Fig. 6B). Interestingly, our microarray analysis of purified splenic B cells from *Tlr7*^−/−^ NOD mice and *Tlr7*^*+/+*^ NOD mice showed that *Tlr7* deficiency significantly decreased the expression of nonclassical MHC-I (MHC-Ib)-encoding genes including *H2-Q6/8* and *H2-Q7/9* in B cells (Fig. 6C), which are Qa2 antigen-encoding genes.^30^ We further confirmed the significantly decreased expression of *H2-Q6/8* and *H2-Q7/9* in *Tlr7*^−/−^ NOD B cells by real-time qPCR (Table 1 and Fig. 6D, E). Although classical MHC-I and MHC-II gene expression was not significantly different between *Tlr7*^*+/+*^ NOD B cells and *Tlr7*^−/−^ NOD B cells (Fig. 6C), the protein expression of the classical MHC-I molecule H2-K^d^ on splenic B cells was significantly reduced in *Tlr7*^−/−^ NOD mice compared with *Tlr7*^*+/+*^ NOD mice (Fig. 6F, G). To investigate the roles of the nonclassical MHC-I molecule Qa2 and the classical MHC-I molecule H2-K^d^ in CD8^+^ T-cell proliferation, we performed a blocking assay in which mitomycin-C-treated wild-type NOD B cells were used as APCs and cocultured with NY8.3 CD8^+^ T cells in the presence of an anti-H2-K^d^ mAb and/or anti-Qa2 mAb. As expected, the anti-H2-K^d^ mAb markedly inhibited the proliferation of NY8.3 CD8^+^ T cells; interestingly, the anti-Qa2 mAb further suppressed the activation of CD8^+^ T cells (Fig. 7A). However, the anti-Qa2 mAb alone did not have a suppressive effect on CD8^+^ T-cell activation (Fig. 7A). When we assessed the blocking ability in culture, the suppressive effect was much greater for both the anti-H2-K^d^ mAb alone and the anti-H2-K^d^ mAb together with the anti-Qa2 mAb when *Tlr7*^−/−^ NOD B cells were used as APCs than when *Tlr7*^*+/+*^ NOD B cells were used as APCs (Fig. 7B, C). In addition to suppressing CD8^+^ T-cell proliferation, blocking Qa2 and H2-K^d^ on B cells simultaneously inhibited CD8^+^ T-cell secretion of proinflammatory TNF-α (Fig. 7D) but not that of IFN-γ and IL-17A (data not shown). Taken together, our studies suggest that *Tlr7* deficiency in NOD mice leads to impaired antigen presentation by B cells to antigen-specific CD8^+^ T cells by reducing the expression of nonclassical and classical MHC-I molecules on B cells.

**Table 1:** Primer information

Genes	Primers	Sequence
*H2-Q6/8*	Forward	5′-CATTATCGTCGGCTACGTGGA-3′
	Reverse	5′-GAGTGTGTGAGAGCCGCC-3′
*H2-Q7/9*	Forward	5′-CATCTCTGTCGGCTACGTGGA-3′
	Reverse	5′-GAGTGTGTGAGAGCCGCC-3′
*GAPDH*	Forward	5′-GGGGTCGTTGATGGCAACA-3′
	Reverse	5′-TGTAGACCATGTAGTTGAGGTCA-3′

## Discussion

Our studies provide novel insights into the role of TLR7 in the context of T1D. We demonstrated that *Tlr7* deficiency suppresses the development of diabetes by altering predominantly B-cell development, differentiation, and functions. Specifically, we found that in the absence of *Tlr7*, B cells expressed increased levels of PD-L1, suppressing CD4^+^ T cells, and the expression levels of both nonclassical MHC-I molecules and classical MHC-I molecules were reduced, limiting the proliferation of antigen-specific CD8^+^ T cells. To our knowledge, this is the first time that nonclassical MHC-I has been shown to be regulated by TLR7; therefore, we have identified a novel mechanism to limit the activation of T cells in autoimmune diabetes and likely in multiple disease settings.

It is known that TLR7, the receptor for ssRNAs including self RNA, plays an important role in modulating the immunopathogenesis of systemic autoimmune disorders, such as SLE.^6,31^ Autoantibodies to nuclear antigens play an essential role in the immunopathogenesis of SLE, a spectrum of systemic autoimmune disorders. In *Tlr7-*deficient mouse models of SLE, disease is ameliorated due to diminished anti-RNA autoantibody levels and suppressed nephritis development.^9^ However, *Tlr7* deficiency does not affect anti-dsDNA autoantibody development, which is TLR9 dependent.^9^ Moreover, studies in both humans with SLE and mouse models of SLE have shown that IFNα also contributes to the immunopathogenesis of this disease.^32–34^ Unlike SLE, T1D is an organ-specific autoimmune disorder mediated mostly by autoreactive T cells. However, the role of TLR7 in T1D was previously unclear. TLR7 has been detected on APCs in the pancreas of patients with newly diagnosed T1D,^35^ suggesting that TLR7 may play a role in the immunopathogenesis of T1D development. Using *Tlr7*-deficient NOD mice, we found that TLR7 contributes to T1D development, as diabetes development was found to be suppressed in NOD mice lacking *Tlr7*. This disease suppression is mediated by alterations in B-cell differentiation and functions. In the absence of *Tlr7*, there were reductions in the number of total B cells in both the BM and peripheral lymphoid tissues. Interestingly, the frequencies of the subsets of marginal zone B cells (CD21^hi^CD23^low^) and Breg cells (CD1d^+^CD5^+^) were significantly increased in the periphery, whereas those of follicular B cells (CD21^low^CD23^hi^) and germinal center B cells (PNA^+^GL-7^+^) were markedly reduced. Importantly, in the absence of *Tlr7*, B cells were hyporeactive to immune stimuli and their antigen presentation to T cells was impaired in vitro. Moreover, *Tlr7*-deficient B cells modulated diabetogenic T cells in vivo. Despite the immunotolerant and regulatory features of *Tlr7-*deficient B cells, surprisingly, these B cells did not produce the immunoregulatory cytokines IL-10 and TGF-β.

Having identified that both autoantibody secretion and antibody-secreting cell frequencies were reduced in *Tlr7*-deficient NOD mice, we investigated the modulatory impact of TLR7 on B-cell interactions with CD4^+^ T cells. *Tlr7*^−/−^ B cells suppressed CD4^+^ T cells in a contact-dependent manner, as evidenced by increased proliferation of *Tlr7*^−/−^ CD4^+^ T cells observed when these cells were cultured in a transwell system that separated the CD4 T cells from direct B-cell contact. This contact-dependent suppression was due to upregulation of PD-L1 expression on *Tlr7*^−/−^ B cells, which directly suppressed diabetogenic CD4^+^ T-cell proliferation, as indicated by the reversal of suppression achieved by blocking PD-L1. This suppression was orchestrated predominantly by changes to PD-L1 on B cells, as PD-1 on CD4^+^ T cells was not altered. Our data support the notion of “checkpoint” inhibition by PD-1/PD-L1 interactions that can limit T-cell proliferation and reduce antibody titers.^36^

To further probe the molecular differences in B cells in the absence of *Tlr7*, we investigated MHC gene expression and demonstrated significant differences in *H2-Q6/8* and *H2-Q7/9*, which are nonclassical MHC class Ib genes, and the expression of these genes was markedly downregulated in *Tlr7*-deficient B cells. We also found that the protein expression of classical MHC-I molecules was reduced on *Tlr7*-deficient B cells. MHC-I plays a critical role in T1D development, and the expression of both classical MHC-I and nonclassical MHC-I molecules is enhanced in the islets of patients with T1D^37^; conversely, MHC-I deficiency is sufficient to prevent β-cell destruction by autoreactive CD8^+^ T cells and T1D development in NOD mice.^38^ Interestingly, we found that *Tlr7*^−/−^ NOD B cells could inhibit the proliferation of cytotoxic CD8^+^ T cells. Thus, our studies provide new insights into the regulation of MHC-I-restricted cytotoxic CD8^+^ T cells in the pathogenesis of T1D by TLR7. It is noteworthy that our study also identified a link between TLR7 and IL-6, as IL-6 expression was significantly reduced in the absence of *Tlr7*.

Although CD4^+^ and CD8^+^ T cells have been demonstrated to play essential roles in the damage to pancreatic β cells occurring during the development of the T1D,^2,27,39–41^ B cells are critical in facilitating T-cell-mediated autoimmunity.^42^ Studies on animal models of human T1D show that B-cell-deficient mice are protected from T1D development, suggesting that B cells are also important in the immunopathogenesis of T1D.^42–45^ Moreover, we previously found that individuals newly diagnosed with T1D had an increased frequency of marginal zone B cells (MZB) but a decreased frequency of follicular B cells (FoB), which are closely associated with altered β-cell function, indicating that B cells are involved in the process of loss of self-tolerance to β cells.^46^ In the current study, we found that *Tlr7*^−/−^ NOD B cells effectively suppressed pathogenic CD4^+^ T-cell responses and inhibited the development of diabetes in a second host. Moreover, we discovered that *Tlr7*^−/−^ NOD B cells could significantly suppress the activation and proliferation of cytotoxic CD8^+^ T cells by regulating the expression of both nonclassical MHC-I molecules and classical MHC-I molecules on B cells. Taken together, our studies show that TLR7 expression on B cells alters their interactions with T cells and thus contributes to the development of T1D. Therefore, targeting TLR7 and/or modulating its function could become an important therapy for T1D.

## Supplementary information

Supplemental materials

## Data Availability

All data generated and analyzed in this study are available from the corresponding author upon request.
