# Supplementary material for: Toll-like receptor 7 deficiency suppresses type 1 diabetes development by modulating B-cell differentiation and function
Source: Cell Mol Immunol. 2021 Jan 11;18(2):328–38. doi: 10.1038/s41423-020-00590-8 (PMC8027372; doi:10.1038/s41423-020-00590-8)
Supplement: Supplementary file 1 — Supplemental materials [file 41423_2020_590_MOESM1_ESM.docx]

**Supplemental materials**

**Figure S1**


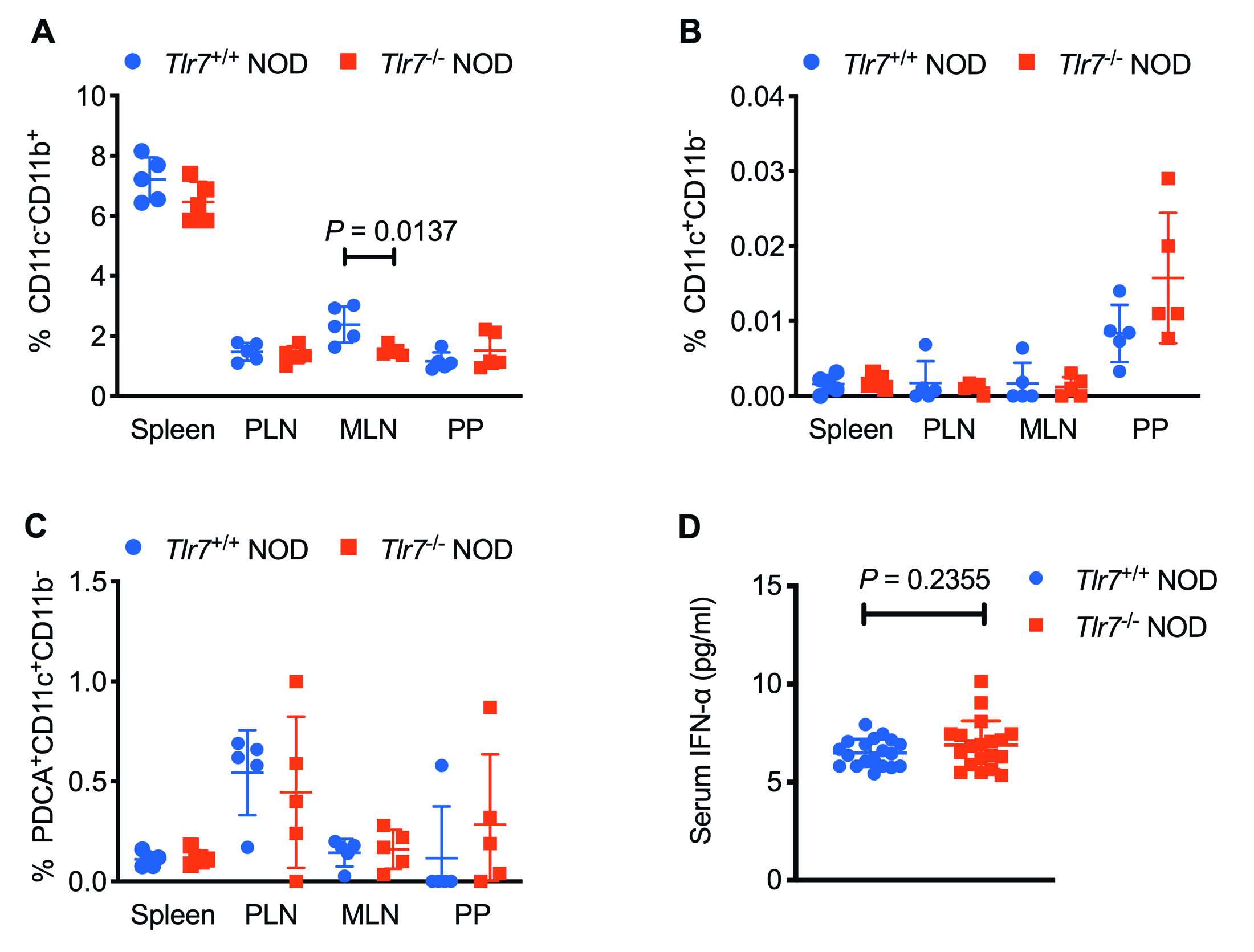


**Figure S1. The proportions of macrophages, cDC and pDC of *Tlr7*^+/+^ and *Tlr7*^-/-^ NOD mice (8-week-old females).** Immune cells were isolated from different lymphoid tissues as indicated and stained with various mAbs for (**A**) Macrophages (CD11c^-^CD11b^+^), (**B**) cDC (CD11c^+^CD11b^-^), and (**C**) pDC (PDCA^+^CD11c^+^CD11b^-^) in *Tlr7*^+/+^ and *Tlr7*^-/-^ NOD mice. The cells were first gated on TCRβ^-^CD19^-^ immune cells. (**D**) Serum concentration of IFN-α in *Tlr7*^+/+^ and *Tlr7*^-/-^ NOD mice. The experiments in (A-C) were performed twice, and consistent results were obtained. Data shown in the figure are from one of the experiment. Data in (D) were pooled from two independent experiments. Data in (A-D) were analyzed by a two-tailed Student's *t*-test, or a two-tailed Mann-Whitney test. PLN, pancreatic lymph node; MLN, mesenteric lymph node; PP, Peyer’s patch.

**Figure S2**


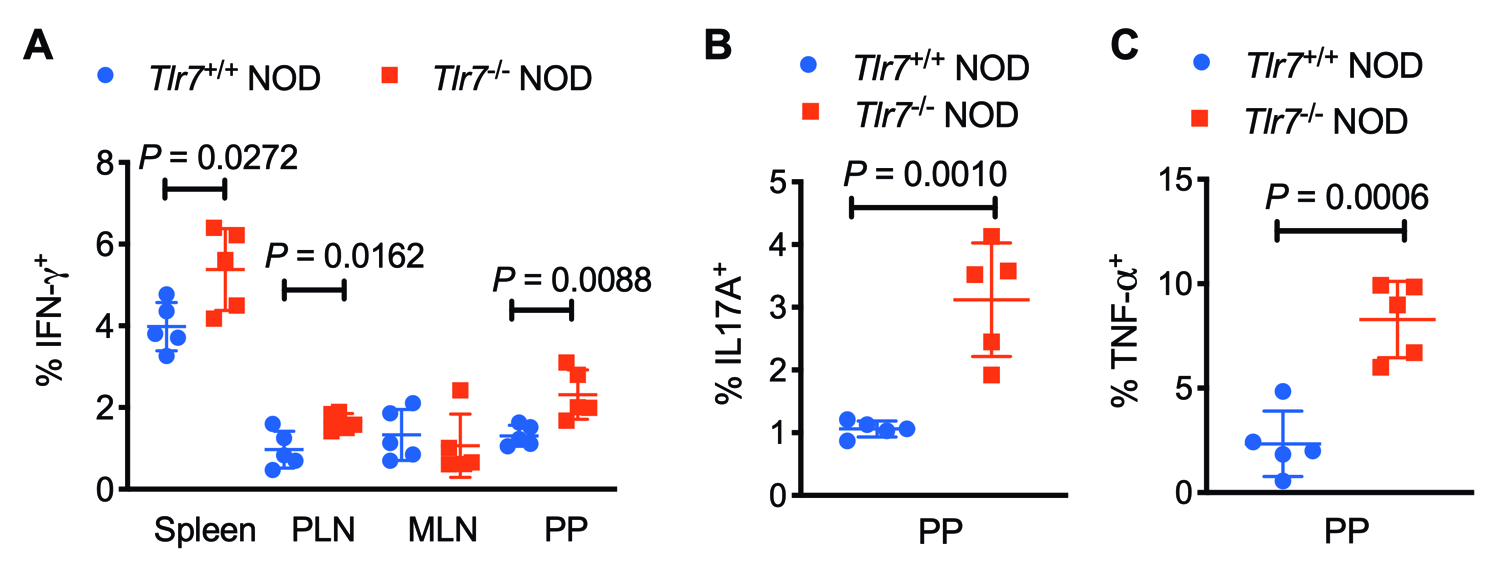


**Figure S2. Expressions of IFN-γ, IL-17A, and TNF-α in CD4^+^ T cells from 8-week-old female *Tlr7*^+/+^ and *Tlr7*^-/-^ NOD mice (8-week-old females, n = 5/group).** (**A**) IFN-γ, (**B**) IL-17A, and (**C**) TNF-α. Immune cells were isolated from different lymphoid tissues as indicated and stained with surface markers as well as various intracellular cytokines prior to flow cytometry analysis. The cells were first gated on TCRβ^+^CD4^+^. The experiments in (A-C) were performed twice, and consistent results were obtained. Data shown in the figure are from one of the experiment. Data were analyzed using a two-tailed Student's t-test and are shown as mean ± SD. PLN, pancreatic lymph node; MLN, mesenteric lymph node; PP, Peyer’s patch.

**Figure S3**


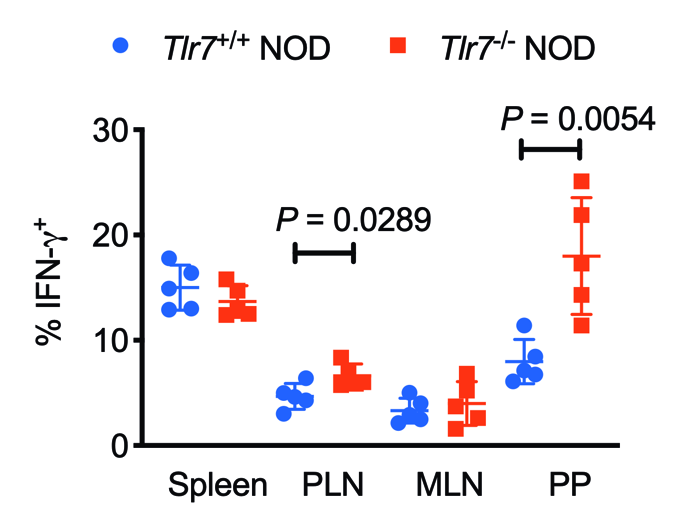


**Figure S3. Expressions of IFN-γ in CD8^+^ T cells from 8-week-old female *Tlr7*^+/+^ and *Tlr7*^-/-^ NOD mice (n = 5/group).** Immune cells were isolated from different lymphoid tissues as indicated and stained with surface markers as well as intracellular IFN-γ prior to flow cytometry analysis. The cells were first gated on TCRβ^+^CD8^+^. The experiment was performed twice, and consistent results were obtained. Data shown in the figure are from one of the experiment. Data were analyzed using a two-tailed Student's t-test and are shown as mean ± SD. PLN, pancreatic lymph node; MLN, mesenteric lymph node; PP, Peyer’s patch.
